# Dynamic interplay between soil microbial communities, enzyme activities, and pear quality across planting years

**DOI:** 10.3389/frmbi.2024.1381270

**Published:** 2024-05-10

**Authors:** Xiaomin Pang, Miao Jia, Ying Zhang, Meihui Chen, Pengyao Miao, Weiting Cheng, Zewei Zhou, Qi Zhang, Jianghua Ye, Jiayu Li, Haibin Wang, Xiaoli Jia

**Affiliations:** ^1^ Center for Information Technology and Laboratory Management, Wuyi University, Wuyishan, China; ^2^ College of Tea and Food Science, Wuyi University, Wuyishan, China; ^3^ College of Horticulture, Fujian Agriculture and Forestry University, Fuzhou, China; ^4^ College of Resources and Environment, Fujian Agriculture and Forestry University, Fuzhou, China; ^5^ College of Life Sciences, Fujian Agriculture and Forestry University, Fuzhou, China; ^6^ Institute of Resources, Environment and Soil Fertilizer, Fujian Academy of Agricultural Sciences/Fujian Key Laboratory of Plant Nutrition and Fertilizer, Fuzhou, China

**Keywords:** pear, planting year, quality, soil enzymes, rhizosphere soil, microbial diversity

## Abstract

Few studies have been reported on the effect of planting years on fruit quality and soil of pear trees. In this study, four planting years (T5, T20, T30, and T40) of Cuiguan pears were used to analyze fruit quality, rhizosphere soil enzymes, and microbial diversity of pear trees, and their correlations. The results showed that the content of sucrose, reducing sugar and ascorbic acid in Cuiguan Pear showed a tendency of increasing and then decreasing with the increase of planting years, in which the highest content was found in 20- and 30-year-old fruits, and the highest content of total acid was found in 5-year-old fruits. Rhizosphere soil enzyme activities varied with planting year, with the highest protease activity in 20-year-old soil, phosphatase and urease in 30-year-old soil, polyphenol oxidase in 5-year-old soil, and sucrase in 40-year-old soil. The microbial diversity index and the number of OTUs showed an increasing and decreasing trend with the increase of planting years. Among the top 11 bacteria in pear rhizosphere soil average relative abundance, with *Bradyrhizobium* decreasing in relative abundance at the peak pear fruiting stages (T20 and T30), while *Acidothermus* showed an increasing trend in relative abundance with increasing planting years. RDA analysis showed that there were differences in the microbial community structure of pear trees at different planting years, and that both sucrose and reducing sugar contents in pears were positively correlated with T20 and T30, ascorbic acid content was positively correlated with T40, whereas the total acid content was positively correlated with T5, and that T20 was positively correlated with soil protease and phosphate mono esterase activities, and that T30 was positively correlated with polyphenol oxidase and urease activities, whereas T40 was positively correlated with sucrase activity. In summary, with the increase of planting years, changes in soil microbial community structure and soil enzyme activity have a significant impact on pear quality formation, and the results of the study provide a theoretical basis for scientific management of pear orchards.

## 1 Introduction

Pear is one of the most important temperate fruit tree species with high economic value (Draga et al., 2023). China is one of the main areas of pear production, and it is the third most important fruit in China (He et al., 2022). Cuiguan pear (*Pyrus pyrifolia*) is a famous early ripening pear variety in China, which is widely planted in southern China (Li et al., 2022). Because of its thin skin, crisp and juicy flesh, fresh taste, thick and sweet juice is loved by people.

The quality of pears is influenced by various aspects, such as planting management practices, growing environment and tree age (Doornbos et al., 2012; Zhang et al., 2020; Musacchi et al., 2021; Liu et al., 2023; Zhang et al., 2023). During growth and development, plants obtain nutrients from the soil while secreting a variety of root secretions. Relevant studies have shown that plant root secretions change during different growth and development periods (Sun et al., 2021). Therefore, this study hypothesized that with the increase of planting years, changes in the root secretion of pear trees altered the soil environment, which in turn affected the uptake of nutrients by pear trees, and ultimately affected the quality formation of pears.

Soil enzymes are important biocatalysts in soils and are very sensitive to environmental factors and are therefore considered important biological indexes of soil quality (Wang et al., 2020). Soil enzymes, which are mainly from microorganisms, are constantly synthesized, accumulated, inactivated and/or decomposed in the soil, while they also play an important role in nutrient cycling and are therefore of great importance to agriculture (Samuel et al., 2008). Chen et al. (2020) showed that long-term cover increased pear soil polyphenol oxidase activity, decreased soil phosphomonoesterase, urease and sucrase activities, and was detrimental to soil nutrient cycling. Kang et al. (2021) showed that the application of organic amendments can significantly increase the activity of soil enzymes related to carbon, nitrogen and phosphorus cycles in pear orchard soils and improve the soil environment. At present, the research on soil enzymes of pear trees mainly focuses on fertilization management and agronomic cultivation, and there is a lack of research on the effect of planting years on soil enzymes of pear trees.

Bacteria growing in the rhizosphere have a significant impact on plant growth, nutrition and health (Oleńska et al., 2020). The aggregation of rhizosphere bacterial communities is the result of selection by plant and soil environmental factors (Mendes et al., 2014). Meanwhile, the composition of the rhizosphere soil bacterial community is the result of long-term competition for nutrients and other resources by plants (Jiang et al., 2017). Pang et al. (2021) has reported that long-term continuous cropping of sugarcane results in a significant decrease in the number of bacteria associated with the function of nitrogen and sulfur cycling in rhizosphere soil, and an increase in the number of pathogenic bacteria. Arafat et al. (2017) found that the rhizosphere bacterial diversity of tea trees decreases significantly with the increase in the number of planting years. Yang et al. (2022) suggested that ancient tea plantations had higher microbial abundance and diversity and a more stable population structure than modern tea plantations. It can be seen that the planting year has a significant effect on soil microbial community structure of the rhizosphere. However, which microorganisms are associated with soil nutrient cycling as planting years increase? And how do these associated microorganisms relate to the fruit quality of pear trees? Based on this, this study took pear trees with different planting years as the research object, and analyzed the effects of planting years on fruit quality, rhizosphere soil enzyme activities, microbial communities and metabolic pathways of pear trees. At the same time, key microorganisms and their metabolic pathways were screened to further analyze the effects of key microorganisms on pear quality formation. The results of the study are expected to provide an important theoretical basis for pear orchard management.

## 2 Materials and methods

### 2.1 Experimental sites

The experimental site was located in Xikou Town, Shouning County, Fujian Province (116°48′34″E, 26°50′38″N). Cuiguan pear trees planted in the mountainous area with 5, 20, 30 and 40 planting years, labeled T5, T20, T30 and T40, respectively, were used for the study. The first pear trees were planted in 1982. The four pear orchards (T5, T20, T30, T40) have a planting area of 11 ha (about 7,200 trees), 20 ha (about 13,000 trees), 18 ha (about 12,000 trees), and 10 ha (about 6,500 trees). The average annual temperature of the four tea plantations is 18.8°C, the rainfall is 1911 mm, and the altitude is 600-650m. Pear trees of four different planting years were consistent in their daily management practices such as fertilization, weeding and watering.

### 2.2 Sample collection

Rhizosphere soil and fruit were collected from pear trees of four planting years using a 5-point sampling method. Briefly, in the pear orchard, a total of five robust pear trees were selected in the east, west, south, north and center, respectively, and excavated the roots of the pear tree with a shovel, shook off the soil on the root with your hand, and collected the soil that fell, that is, rhizosphere soil (shake-down method). After picking out the roots remaining in the rhizosphere soil, the soil was mixed and placed in an ice box and brought back to the laboratory to be stored in a -80°C refrigerator for subsequent index measurements. At the same time, two pears were randomly selected from each of the five pear trees, and a total of 10 pears were collected for quality index determination in each planting year.

### 2.3 Fruit quality analysis

Fruit quality was determined by sucrose, reducing sugar, ascorbic acid and total acid content. Sucrose content was determined with reference to GB5009.8-2016 (GAQS, 2016b), pears were crushed and mixed, 10 g of homogenized liquid was taken to a 100 mL colorimetric tube, 50 mL of distilled water was added, 5 mL of zinc acetate solution and 5 mL of potassium ferricyanide solution were added, mixed well and ultrasonically for 30 min, and then fixed volume to 100 mL. The fixed solution was filtered and then determined using high performance liquid chromatography for sucrose content. Reducing sugar content was determined with reference to GB5009.7-2016 (GAQS, 2016a), pears were crushed and mixed, 25 g of homogenized liquid was taken into a 250 mL volumetric flask, 50 mL of distilled water was added, mixed, and then 10 mL of alkaline copper tartrate solution and 4 mL of sodium hydroxide solution were added, mixed, and fixed. Allow to stand for 30 minutes and then filter. The filtrate was used to determine reducing sugar content by potassium permanganate titration. Ascorbic acid (Vc) content was determined with reference to GB5009.86-2016 (GAQS, 2016c), pears were crushed and mixed, and 2 g homogenized liquid was taken into a 50 mL volumetric flask and fixed to 50 mL with 20 g/L metaphosphoric acid solution. All the solution was transferred to a centrifuge tube and sonicated for 5 min, then centrifuged at 4000 r/min for 5 min, and the supernatant was taken through a 0.45 μm filter membrane, and the Vc content in the filtrate was determined using high performance liquid chromatography. The total acid content was determined with reference to GBT12456-2008 (GAQS, 2008), the pears were crushed and mixed, 200 g of homogenized liquid was taken and mixed with equal amount of water. 10 g of the above solution was taken and transferred to a 250 mL volumetric flask with distilled water at 80°C, followed by a boiling water bath for 30 min, removed, cooled to room temperature and then concentrated to 250 mL. The solution was filtered and the filtrate was titrated with a 0.1 mol/L standard solution of sodium hydroxide.

### 2.4 Determination of soil enzyme activity

Soil samples were mixed well, passed through a 2 mm sieve, and soil enzyme activity was determined using Li et al. (2008). Determination of protease activity: 5 g of fresh soil was added to 25 mL of casein matrix solution (5 g/L), then 1 mL of toluene was added and the solution was incubated at 38°C for 48 h. Then, 25 mL of 10% trichloroacetic acid was added, and the solution was allowed to stand for 30 min and then filtered. Folin colorimetry was used and the absorbance at 680 nm was measured. The experiment was repeated three times. Determination of polyphenol oxidase activity: 1 g of soil was added to 4 mL of citrate-phosphate buffer (pH 4.5), followed by 10 mL of 1% pyrogallol and mixed well. The solution was incubated at 30°C for 2 h. After incubation, 35 mL of ether was added and the solution was shaken for 15 min at room temperature and then left to stand. The absorbance of the ether phase was measured at 430 nm. The experiment was repeated three times. Determination of phosphomonoesterase activity: 0.2 mL of toluene, 4 mL of acid phosphatase buffer (pH 6.5) and 1 mL of 4-nitrophenyl disodium phosphate solution were added to 1 g of fresh soil. The solution was incubated at 37°C for 1 h. Then 1 mL of 0.5 mol/L calcium chloride and 4 mL of 0.5 mol/L sodium hydroxide were added, mixed well, and centrifuged at 4000 r/min for 5 min, and the absorbance of the supernatant was measured at 400 nm. Determination of urease activity: 2 mL of toluene was added to 10 g of soil and left to stand for 15 min, then 10 mL of urea solution and 20 mL of citrate buffer (pH 6.7) were added and mixed well. After incubation at 38°C for 3 h, the solution was fixed to 100 mL with distilled water and filtered. Then 1 mL of filtrate was added to 9 mL of distilled water and 4 mL of sodium phenol. Finally, 3 mL of sodium chlorate solution was added and the solution was made up to 50 mL. The solution was allowed to stand for 20 min and then the absorbance of the solution was measured at 578 nm. The experiment was repeated three times. Determination of sucrase activity: 5 g of fresh soil was added to a beaker containing 15 mL of 8% sucrose solution, 5 mL of phosphate buffer (pH 5.5) and 5 drops of toluene. The solution was mixed homogeneously and incubated at 37°C for 24 h. Next, the solution was filtered and 3 mL of 0.5% 3,5-dinitrosalicylic acid was added to 1 mL of the filtrate. The solution was incubated in a boiling water bath for 5 min, then cooled to 25 mL. The absorbance was measured at 508 nm. The experiment was repeated three times.

### 2.5 Soil microbial diversity analysis

#### 2.5.1 Soil DNA extraction and sequencing

Soil DNA was extracted using MoBio PowerSoil DNA isolation kit (Carlsbad, CA, USA). DNA samples were tested for quality by 1% agarose gel electrophoresis and spectrophotometry. Qualified DNA samples were stored at -20°C for subsequent use. The primers for amplification of the highly variable V3-V4 region of the bacterial 16S rRNA gene were 338F(5’-ACTCCTACGGGAGGCAGCAG-3’) and 806R(5’-GGACTACHVGGGTWTCTAAT-3’) (Fadrosh et al., 2014). PCR reaction system was 25μL, including 12.5μL of 2× Taq PCR MasterMix, 3μL of BSA (2 ng/μL), 2μL of Primer (5μM), 2μL of template DNA, and 5.5μL of ddH2O. The PCR reaction program was as follows: pre-denaturation at 95°C for 5 min; denaturation at 95°C for 45 s, annealing at 55°C for 50 s, extension at 72°C for 45 s, 32 cycles, and finally, extension at 72°C for 10 min (Yao et al., 2014). Each sample was repeated three times and the PCR products from the same sample were mixed and detected by 2% agarose gel electrophoresis. PCR products were recovered using the QIAquick Gel Extraction Kit (QIAGEN, Germany) and then eluted with Tris-HCl for quantification using RT-PCR. Sequencing data are stored in NCBI’s Sequence Read Archive (SRA) under accession number PRJNA1055603.

#### 2.5.2 Bioinformatics analysis

Raw data were first screened and sequences were removed from consideration if they were shorter than 230 bp, had a low quality score (≤20), contained ambiguous bases or did not exactly match primer sequences and barcode tags, and were separated using sample-specific barcode sequences. Qualified reads were clustered into operational taxonomic units (OTUs) at a 97% similarity level (Edgar, 2013) using the Uparse algorithm of Vsearch (v2.7.1) software. The BLAST tool was used to classify all sequences into different taxonomic groups against the Silva138 database (Pruesse et al., 2007; Willis, 2019).

QIIME (v1.8.0) was used to generate rarefaction curves and calculate richness and diversity indices based on OTU information. To compare community membership and structure in different samples, a heatmap was generated with the top 20 OTUs using Mothur (Jami et al., 2013). Based on the results of taxonomic annotation and relative abundance, R (v3.6.0) software was used for bar-plot diagram analysis. To examine the similarity between different samples, clustering analysis and PCA were analyzed by R (v3.6.0) based on OTU information from each sample (Wang et al., 2012). The evolutionary distances between microbial communities from each sample were calculated using Bray Curtis algorithms and represented as an Unweighted Pair Group Method with Arithmetic Mean (UPGMA) clustering tree describing the dissimilarity (1-similarity) between multiple samples (Jiang et al., 2013). A Newick-formatted tree file was generated through this analysis. Alpha diversity was applied to analyze microbial diversity, including Chao1, Shannon, and Simpson, which were calculated with Mothur software (version 1.30.1, http://www.mothur.org/). A beta-diversity analysis based on the Bray-Curtis dissimilarity matrix was calculated using R 2.13.2 (R Development Core Team) software.

### 2.6 Data analysis

Variance analysis (ANOVA) and significance analysis were performed using SPSS 20.0 software, and the least significant difference (LSD) test was performed. The relationship between fruit quality and soil enzyme activity was analyzed by Pearson’s Correlation Heatmap using the pheatmap package. Orthogonal partial least squares-discriminant analysis (OPLS-DA) based on nonlinear iterative partial least squares was calculated using R 2.13.2 (R Development Core Team) software. Redundancy analysis (RDA) based on Monte Carlo substitution was performed using R 2.13.2 (R Development Core Team) software.

## 3 Results

### 3.1 Fruit quality analysis

**Figure 1:**
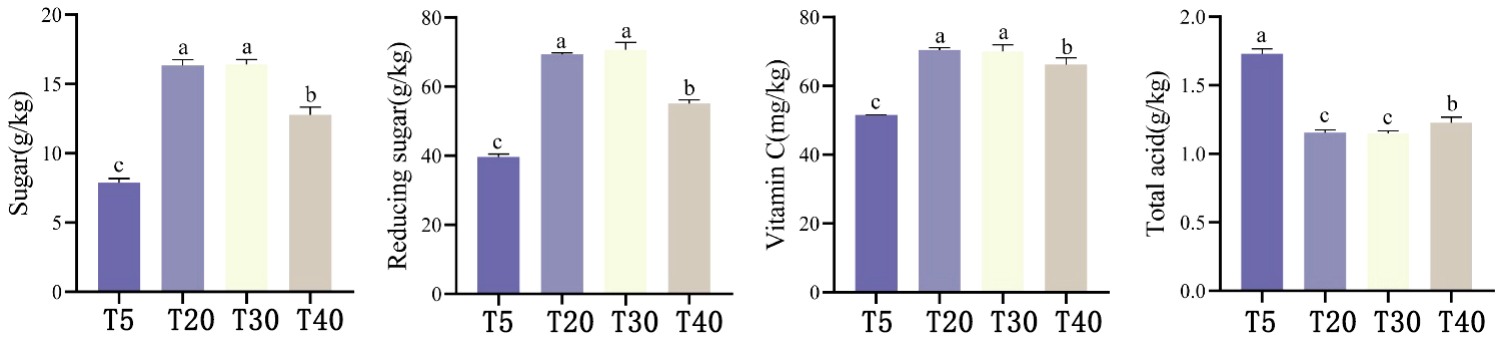
Effect of different planting years on pear quality. T5: 5 planting year, T20: 20 planting year, T30: 30 planting year, T40: 40 planting year. Different lowercase letters represent significant differences at *p* < 0.05.

Analysis of the effect of planting years on the quality of pears showed that (**Figure 1**), with the increase of planting years, sucrose, reducing sugar and Vc content in pears showed a trend of first increasing and then decreasing, which was manifested as T30 ≈ T20 > T40 > T5. T30 and T20 had the highest sucrose, reducing sugar and Vc contents all were significantly higher (*p*<0.05) than T40 and T5. Sucrose, reducing sugar and Vc contents of T40 were significantly higher (*p*<0.05) than those of T5. Total acid content showed T5 > T40 > T20≈ T30, where T5 had the highest total acid content and was significantly higher than the other planting years (*p*<0.05), whereas total acid content of T40 was again significantly higher than that of T20 and T30 (*p*<0.05). It can be seen that the quality of pears is optimal and stable during the peak fruiting period (T20 and T30).

### 3.2 Soil enzyme activity analysis

**Figure 2:**
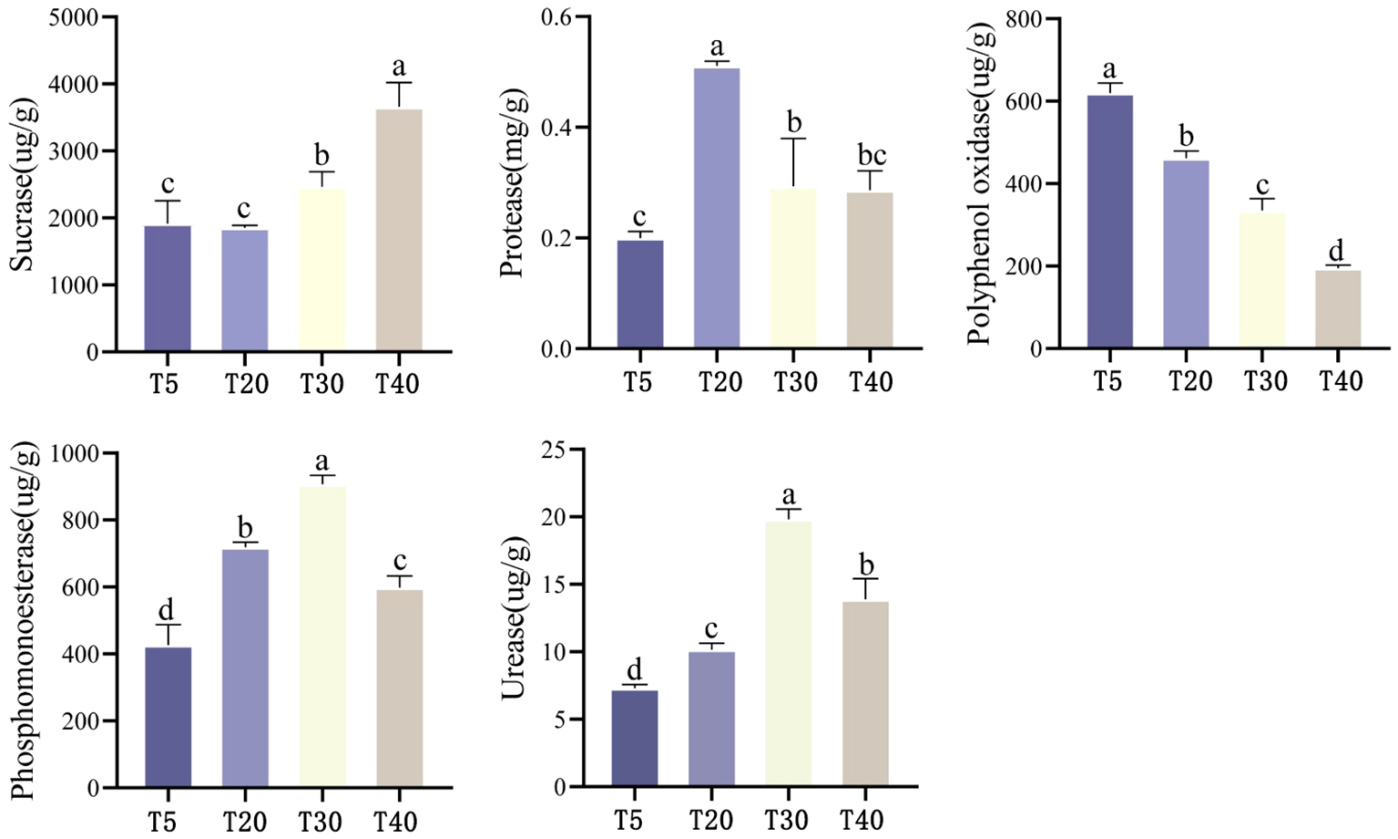
Effect of different planting years on enzyme activity in rhizosphere soil of pear tree. T5: 5 planting year, T20: 20 planting year, T30: 30 planting year, T40: 40 planting year. Different lowercase letters represent significant differences at *p* < 0.05.

The effect of planting years on soil enzyme activity of pear trees showed (**Figure 2**) that soil sucrase activity tended to increase with the increase of planting years, which was manifested as T40 > T30 > T5 ≈ T20, and the differences between planting years reached the level of significance except for the difference between T5 and T20 (*p*<0.05). With the increase of planting years, soil protease activity showed an increasing and then decreasing trend, which was T20 > T30 ≈ T40 > T5. The highest protease activity was found in T20, which was significantly higher than that of T5, T30 and T40 (*p*<0.05). Soil polyphenol oxidase activity showed a decreasing trend with increasing planting years, which was manifested as T5>T20>T30>T40, and there was a significant difference (*p*<0.05) between all planting years. Soil phosphomonoesterase activity showed an increasing trend with increasing planting years, which was manifested as T30>T20>T40>T5, and there were significant differences (*p*<0.05) among all planting years. Soil urease activity showed an increasing trend with increasing planting years, which was manifested as T30>T40>T20>T5, and there were significant differences (*p*<0.05) among all planting years. It can be seen that soil enzyme activities related to the cycling of nitrogen and phosphorus were significantly increased in pear tree soils during the peak fruiting period (T20 and T30), favoring the cycling of these two elements.

### 3.3 Heatmap analysis of correlation networks

**Figure 3:**
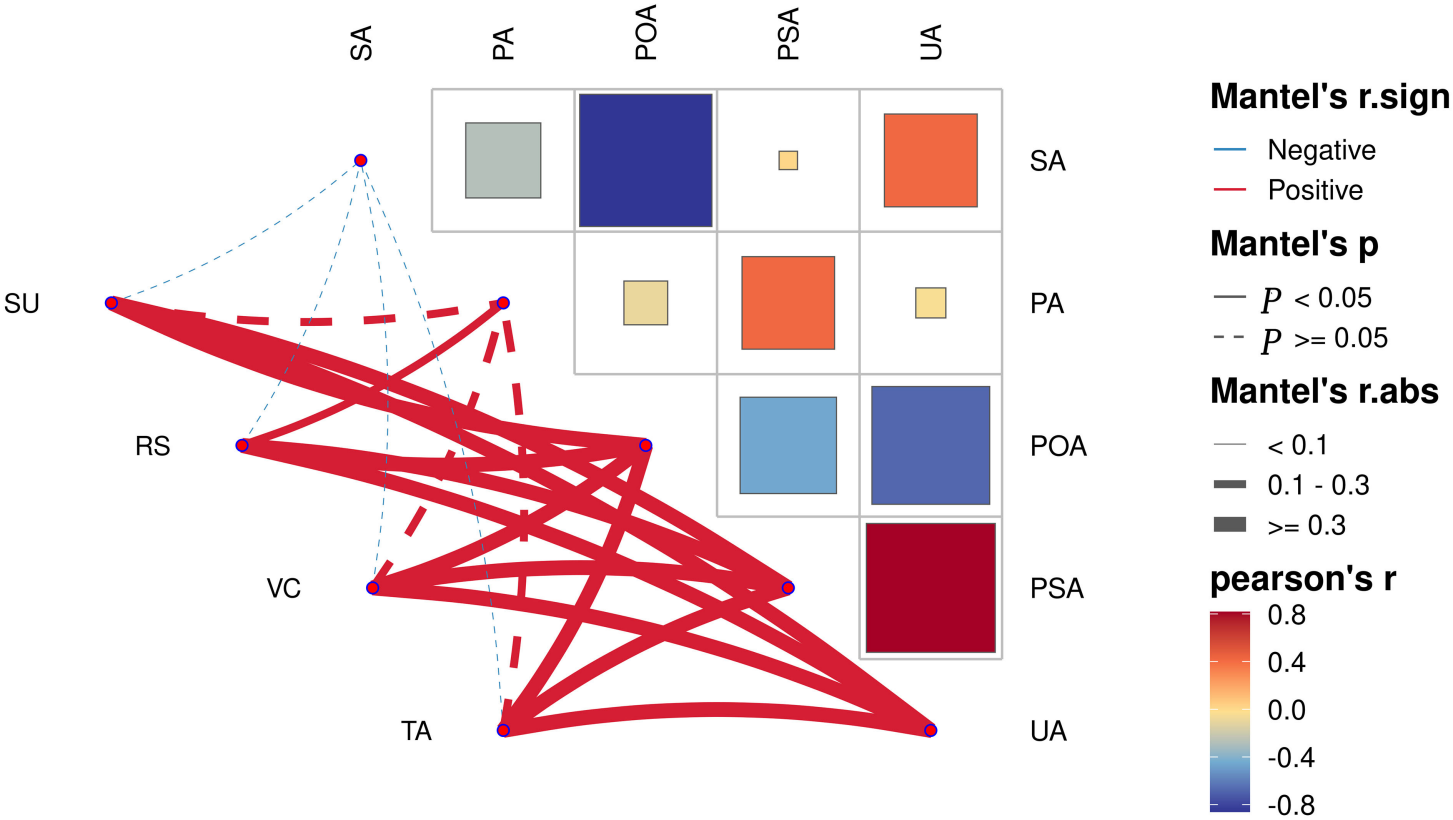
Analysis of correlation network between pear fruit quality and soil enzyme in different planting years. SA, sucrase; PA, protease; POA, polyphenol oxidase; PSA, phosphomonoesterase; UA, urease; SU, sugar; RS, seducing sugar; VC, vitamin C; TA, total acid.

Correlation analysis of soil enzyme activity at different planting years showed (**Figure 3**) that urease and phosphomonoesterase were significantly positively correlated (*p*<0.05). Polyphenol oxidase and sucrase were significantly negatively correlated (*p*<0.05). Analysis of the correlation between fruit quality indexes and soil enzyme activity at different planting years showed (**Figure 3**) that polyphenol oxidase, phosphomonoesterase and urease were all significantly positively correlated (*p*<0.05) with four quality indexes (sucrose, reducing sugar, ascorbic acid and total acid). Protease was significantly and positively correlated (*p*<0.05) with reducing sugar. Sucrase was negatively correlated with the four quality indexes, but none was significant. It can be seen that soil enzyme activities were closely related to fruit quality indexes.

### 3.4 Soil microbial diversity analysis

**Figure 4:**
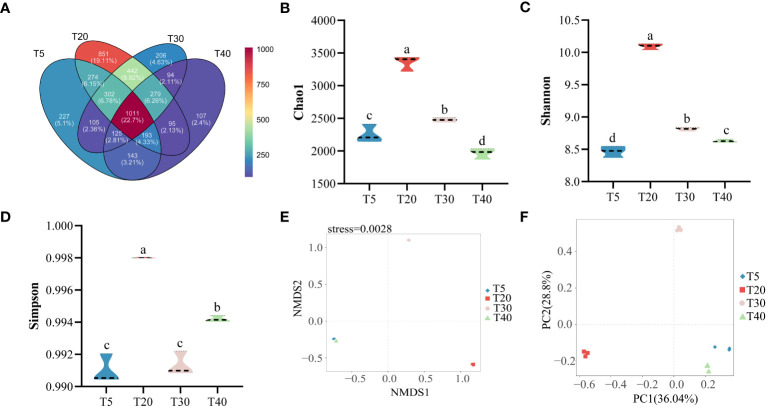
Rhizosphere soil microbial diversity of pear trees with different planting years. T5: 5 planting year, T20: 20 planting year, T30: 30 planting year, T40: 40 planting year. **(A)** Analysis of the number and similarity of OTUs detected in the rhizosphere soils of pear trees with different planting years; **(B)** Analysis of the chao1 diversity index of rhizosphere soil OTUs of pear trees with different planting years; **(C)** Analysis of the Shannon index of rhizosphere soil OTUs of pear trees with different planting years; **(D)** Analysis of the simpson diversity index of rhizosphere soil OTUs of pear trees with different planting years; **(E)** NMDS analysis of the β-diversity index of rhizosphere soil OTUs of pear trees with different planting years; **(F)** PCA analysis of rhizosphere soil OTUs of pear trees with different planting years. Different lowercase letters represent significant differences at *p* < 0.05.

Sequencing results of the rhizosphere soil bacterial community at different planting years showed (**Figure 4A**) that a total of 4488 OTUs were detected in all samples, and 4454 were obtained after rarefaction. An average of 1767, 2767, 1927, and 1522 OTUs were detected in T5, T20, T30, and T40, respectively (**Supplementary Table S1**). There were 1100 OTUs common to the four planting years, accounting for 22.70% of the total OTUs. Further analysis found that 227, 851, 206, and 107 OTUs were specific to T5, T20, T30, and T40, respectively, accounting for 5.10%, 19.11%, 4.63%, and 2.40% of the total OTUs, respectively. The α-diversity analysis found that the chao1 index showed an increase and then a decrease with increasing planting years, which was manifested as T20>T30>T5>T40, with significant differences (*p*<0.05) among all four planting years (**Figure 4B**). The Shannon index showed an increase and then a decrease with increasing planting years, as shown by T20>T30>T40>T5, with significant differences (*p*<0.05) in all four planting years (**Figure 4C**). The Simpson index showed an increase followed by a decrease and then an increase with the increase in planting years, which was manifested as T20>T40>T30>T5, where T20 was the largest and significantly higher than the other planting years (*p*<0.05), and T40 was significantly higher than T30 and T5 (*p*<0.05) (**Figure 4D**). Based on β-diversity, analysis of the complexity of the soil bacterial community in the rhizosphere of pear trees using the NMDS method showed (**Figure 4E**) that the soil microorganisms of different planting years could be clearly differentiated (NMDS: stress = 0.0028, PERMANOVA, *p* < 0.01). PCA analysis of the rhizosphere soil bacterial community of pear trees showed (**Figure 4F**) that the two principal components effectively differentiated between the four planting years, and their overall contribution was 64.84%. It can be seen that the planting year had a significant effect on the richness and community structure of the rhizosphere soil bacterial community of pear trees.

### 3.5 Screening for key microorganisms

**Figure 5:**
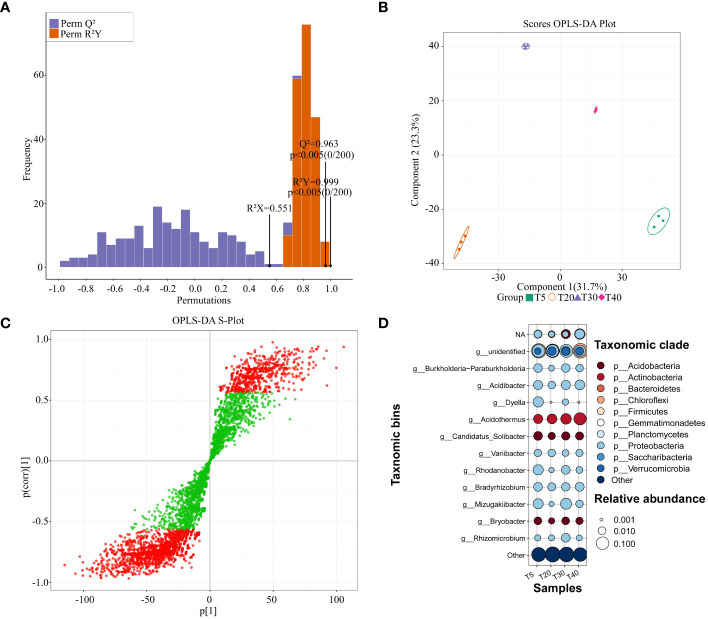
Screening of key differentiating bacteria in rhizosphere soil of pear trees with different planting years. T5: 5 planting year, T20: 20 planting year, T30: 30 planting year, T40: 40 planting year; **(A)** Test plot of OPLS-DA model for rhizosphere soils of pear trees with different planting years; **(B)** Scores OPLS-DA plot for analysis of within- and between-group differences in rhizosphere soils of pear trees with different planting years; **(C)** OPLS-DA S-Plot for screening of key differential OTUs in rhizosphere soils of pear trees with different planting years, Green indicates that the VIP absolute value is less than 1, and red indicates that the vip absolute value is greater than 1. **(D)** Bubble map analysis of key differentiating bacteria in rhizosphere soil of pear trees with different planting years.

Based on the abundance of rhizosphere soil bacterial OTUs, the OPLS-DA model was used to screen for key bacteria that varied significantly between planting years (**Figure 5**). The results showed that the OPLS-DA model for rhizosphere soil bacteria of pear trees at different planting years had a goodness of fit R^2^Y value of 0.999 (*p* < 0.005) and a predictability Q^2^ value of 0.963 (*p* < 0.005) (**Figure 5A**). It can be seen that the R^2^Y and Q^2^ values of the model have reached a significant level, and the model has a good fit and high credibility for further analysis. The scores of the OPLS-DA plot showed (**Figure 5B**) that OPLS-DA effectively distinguished samples from different planting years in different regions. S-plot analysis showed (**Figure 5C**) that there were 2193 key OTUs (VIP >1) that distinguished between samples from different planting years. Significant differences were observed in rhizosphere soil bacteria of pear trees in different planting years (**Supplementary Table S2**). Based on the obtained key OTUs, the corresponding bacteria were matched and categorized for analysis (**Figure 5D**). After removing the mismatched bacteria (others), the remaining bacteria can be categorized into 11 genera including *Bradyrhizobium*, *Burkholderia-Paraburkholderia*, *Acidibacter*, *Dyella*, *Acidothermus*, *Candidatus_Solibacter*, *Variibacter*, *Rhodanobacter*, *Mizugakiibacter*, *Bryobacter*, and *Rhizomicrobium*, with which they correspond to 10 phyla respectively Proteobacteria, Chloroflexi, Acidobacteria, Actinobacteria, Firmicutes, Bacteroidetes, Planctomycetes, Gemmatimonadetes and Verrucomicrobia. With the increase of planting years, at the genus level, the relative abundance of *Bradyrhizobium* declined at the peak fruiting period (T20 and T30), the relative abundance of *Acidothermus* increased, and there was no pattern of change for other bacteria. At the phylum level, the relative abundance of Acidobacteria, Gemmatimonadetes, and Verrucomicrobia increased during the peak fruiting period. It can be found that the relative abundance of rhizosphere soil bacteria changed significantly between planting years, which may affect the fruit quality of pear trees.

### 3.6 Interaction analysis

**Figure 6:**
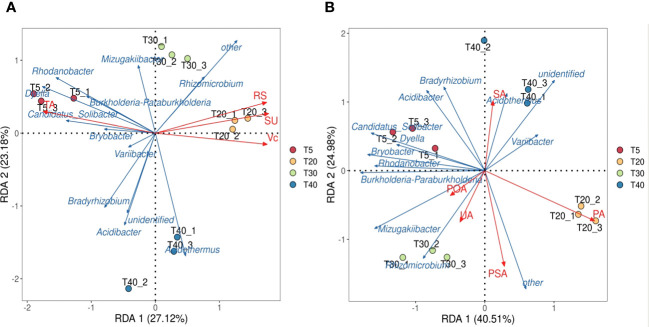
Interaction analysis of key genera of microorganisms with quality index of pear and soil enzyme. T5, 5 planting year; T20, 20 planting year; T30, 30 planting year; T40, 40 planting year; SA, sucrase; PA, protease; POA, polyphenol oxidase; PSA, phosphomonoesterase; UA, urease; SU, sugar; RS, Reducing sugar; VC, vitamin C; TA, total acid. **(A)** Redundancy analysis of key genera of microorganisms with quality index of pear; **(B)** Redundancy analysis of key genera of microorganisms with rhizosphere soil enzyme of pear tree.

On the basis of these analysis, the interactions of key microbial genera of rhizosphere soils with quality index contents and soil enzyme activities of pear trees were further analyzed. Redundancy analysis of key soil microbial genera and pear quality indexes showed (**Figure 6A**) that both sucrose and reducing sugar were positively correlated with T20 and T30, ascorbic acid was positively correlated with T40, and total acid was positively correlated with T5. Redundancy analysis of key microbial genera and soil enzymes showed (**Figure 6B**) that T20 was positively correlated with protease and phosphomonoesterase, T30 was positively correlated with polyphenol oxidase and urease, and T40 was positively correlated with sucrase. It can be seen that significant changes in soil bacterial community abundance occurred when pear trees entered the peak fruiting period, which increased soil enzyme activity and promoted the formation of fruit quality.

## 4 Discussion

### 4.1 Quality of pears of different planting years

Pear is an economically important fruit that is widely cultivated in China. At present, the influencing factors on pear quality formation mainly include cultivation and management methods, soil environment, and post-harvest storage (Zhang et al., 2020, 2023; Adhikary et al., 2021a; Adhikary et al., 2021b), while the effect of planting years on pear quality formation has rarely been reported. The results of this study showed (**Figure 1**) that the sweetness (sucrose and reducing sugar) of pears tended to increase and then decrease with the increase of planting years, and the acidity of pears was highest in young age. Ahmed and Dennis (1992) found that the fruit of young trees contains higher levels of anti-aging hormones (growth hormones and gibberellins), which may delay the ripening process and prevent the conversion of organic acids in large quantities, and as a result, the fruit acidity of young trees is higher than that of fruit trees of other ages.

### 4.2 Rhizosphere soil enzymes of pear trees of different planting years

Soil enzymes play an important role in maintaining soil ecology and health. Proteases play an important role in soil nitrogen transformation and plant nitrogen nutrition, phosphomonoesterase catalyzes the production of free phosphate groups by breaking the monoester bond in phosphate monoester compounds, urease specifically catalyzes urea hydrolysis to release ammonia and carbon dioxide, sucrase plays an important role in increasing soil labile nutrients, and polyphenol oxidase oxidizes soil aromatic compounds, thus facilitating their cycling (Neemisha and Sharma, 2022). Zhao et al. (2023) showed that soil phosphatase activity increased significantly and then decreased with the increase in the number of years of planting tomato in the facility. Zydlik et al. (2023) showed that adding biochar to apple orchards could increase soil protease activity and improve the soil environment. Ren et al. (2021) showed that partial replacement of chemical fertilizers with organic fertilizers could increase rhizosphere soil urease, sucrase, and phosphatase activities in maize. In this study, rhizosphere soil protease, urease and phosphatase activities of pear trees were significantly increased during the peak fruiting season (**Figure 2**), which was beneficial for soil nutrient cycling. Matocha et al. (2004) reported that the application of chemical fertilizers inhibited polyphenol oxidase activity in agricultural soils. The results of this study also revealed a decreasing trend in polyphenol oxidase activity with increasing planting years, which may be due to the accumulation of chemical fertilizers in soils cultivated for many years, thus inhibiting polyphenol oxidase activity. Correlation results showed that polyphenol oxidase, urease and phosphatase activities were positively correlated with the quality indexes of pears (**Figure 3**), indicating that soil enzyme activities were closely related to the quality of pears.

### 4.3 Microbial diversity of rhizosphere soils of pear trees of different planting years

Bacteria in the rhizosphere soil of plants have a profound effect on their growth, nutrient uptake, and health (Oleńska et al., 2020). Wan et al. (2019) showed that crop yield declines in acidic soils may be related to the attenuation of the function of the rhizosphere bacterial community. Ren et al. (2020) investigated the effects of long-term continuous nitrogen application on wheat yield and rhizosphere soil microorganisms and showed that long-term continuous nitrogen application reduces the number and diversity of OTUs of bacteria, affects soil nutrient cycling, and ultimately leads to reduced wheat yield. Rhizosphere soil microorganisms of plants are highly sensitive and respond rapidly to external changes in the environment, especially with changes in the soil environment, but fewer studies have been reported on the relationship between different planting years and rhizosphere microbial communities of fruit trees. Qiang et al. (2020) analyzed soil microbial communities in citrus orchards with different planting years and showed that planting years significantly affected the community composition of soil microorganisms. This study showed that rhizosphere soil microbial diversity of pear trees first increased and then decreased with increasing planting years (**Figure 4**). This is consistent with Pang et al. (2024) results that the utilization rate of carbon source by rhizosphere soil microorganisms of pear trees also increases first and then decreases with increasing planting years. *Bradyrhizobium* is closely related to plant nitrogen fixation and can increase plant nitrogen fixation capacity (Akley et al., 2022). It may be that pear trees are not part of the legume family, which is not conducive to the propagation of *Bradyrhizobium*, and that the relative abundance of other microorganisms increases and the relative abundance of *Bradyrhizobium* decreases during the peak fruiting period. *Acidothermus* can grow under acidic conditions and degrade plant residues (Kim et al., 2016). *Acidothermus* aggregation promotes increases in soil organic matter and magnesium, as well as available nitrogen and calcium (Steven et al., 2021). When pear trees entered the peak fruiting period, their secretion and apomixis increased, which was conducive to the propagation of *Acidothermus*, increasing nutrients in the soil, and favoring the formation of fruit quality (**Figure 5D**). RDA analysis showed that the aggregation of soil microorganisms in the rhizosphere of pear trees facilitated phosphatase and protease activities during the peak fruiting period, and at the same time promoted sugar accumulation in fruits.

## 5 Conclusions

**Figure 7:**
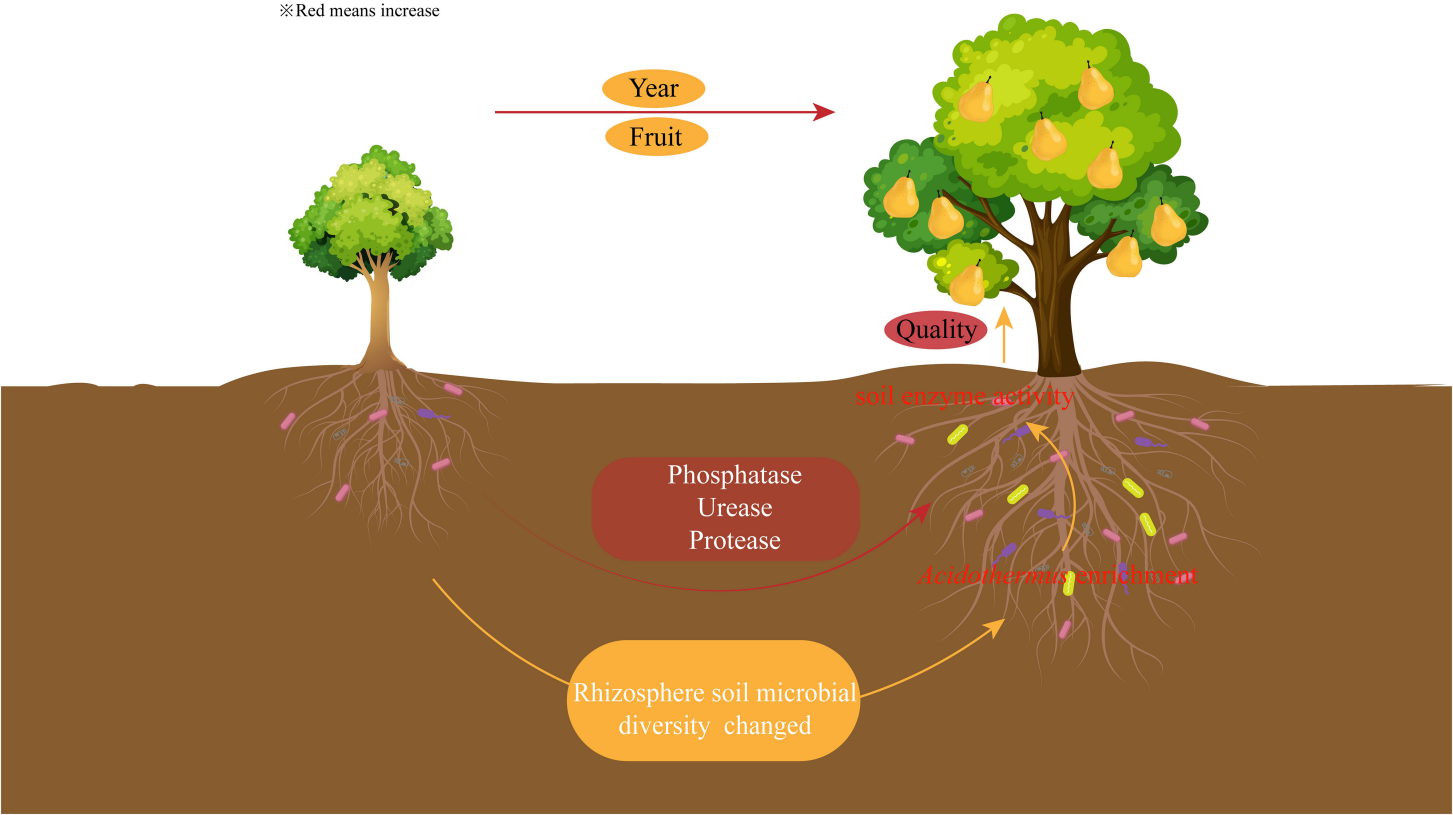
Effect of planting years on soil enzyme and microbial diversity and fruit quality of pear trees.

There were significant differences in the quality of pears between different planting years, with the sweetest pears at the peak fruiting period. The rhizosphere soil enzyme activities of pear trees changed with the increase of planting years, and phosphatase, urease and protease activities were significantly increased during the peak fruiting period. The microbial diversity of rhizosphere soil also changed with the increase of planting years, and the microbial diversity and the number of OTUs were higher in the peak fruiting period than in other periods. The enrichment of *Acidothermus* during the peak fruiting period favored nutrient cycling in rhizosphere soil of pear trees, enhanced soil enzyme activity, and promoted pear quality formation (**Figure 7**). It is suggested that pear quality can be improved by regulating microorganisms and enzyme activity during pear planting.

## Data availability statement

The datasets presented in this study can be found in online repositories. The names of the repository/repositories and accession number(s) can be found below: https://www.ncbi.nlm.nih.gov/, PRJNA1055603.

## Author contributions

XP: Conceptualization, Data curation, Formal analysis, Methodology, Software, Writing – original draft, Writing – review & editing. MJ: Investigation, Methodology, Software, Validation, Writing – original draft, Writing – review & editing. YZ: Conceptualization, Methodology, Software, Writing – original draft. MC: Formal analysis, Methodology, Writing – original draft. PM: Data curation, Methodology, Software, Writing – original draft. WC: Conceptualization, Investigation, Software, Writing – original draft. ZZ: Methodology, Software, Writing – original draft. QZ: Funding acquisition, Supervision, Writing – original draft. JY: Funding acquisition, Resources, Writing – original draft. JL: Funding acquisition, Investigation, Writing – original draft. HW: Funding acquisition, Software, Writing – original draft, Writing – review & editing. XJ: Conceptualization, Funding acquisition, Resources, Writing – original draft, Writing – review & editing.
