# Supplementary material for: Dynamic interplay between soil microbial communities, enzyme activities, and pear quality across planting years
Source: Front Microbiomes. 2024 May 10;3:1381270. doi: 10.3389/frmbi.2024.1381270 (PMC12993529; doi:10.3389/frmbi.2024.1381270)
Supplement: Supplementary file 1 [file Table_1.docx]

Table S1 The number of OTUs in the samples

| SampleID | Final_tags | OTUs |
| --- | --- | --- |
| 5-years | 24810 | 1767±71 |
| 20-years | 24810 | 2767±70 |
| 30-years | 24810 | 1927±22 |
| 40-years | 24810 | 1522±33 |

Values are means ± S.E. from 3 replicates.

Table S2 Different rhizosphere soil microorganisms of pear trees with different planting years

| Treatment | Acidothermus | Mizugakiibacter | Acidibacter | Bradyrhizobium | Rhodanobacter | Candidatus_Solibacter | Burkholderia-Paraburkholderia | Dyella | Bryobacter | Variibacter | Rhizomicrobium |
| --- | --- | --- | --- | --- | --- | --- | --- | --- | --- | --- | --- |
| T5 | 0.0196±0.0015c | 0.0314±0.0029b | 0.0191±0.0032b | 0.0163±0.0001a | 0.0320±0.0025a | 0.0172±0.0011a | 0.0156±0.0003a | 0.0343±0.0023a | 0.0098±0.0001a | 0.0083±0.0001a | 0.0046±0.0001b |
| T20 | 0.0268±0.002c | 0.0029±0.0004d | 0.0092±0.0020c | 0.0118±0.0027a | 0.0031±0.0008c | 0.0081±0.0003c | 0.0042±0.0007c | 0.0007±0.0001b | 0.0048±0.0001b | 0.0091±0.0001a | 0.0042±0.0001b |
| T30 | 0.0391±0.0009b | 0.0492±0.0027a | 0.0198±0.0019b | 0.0138±0.0001a | 0.0166±0.0026b | 0.0131±0.0013ab | 0.0147±0.0013a | 0.0052±0.0001b | 0.0100±0.0014a | 0.0062±0.0001a | 0.0166±0.0011a |
| T40 | 0.0872±0.0046a | 0.0086±0.0006c | 0.02768±0.0029a | 0.0175±0.0031a | 0.0061±0.0034c | 0.0119±0.0017b | 0.0089±0.0008b | 0.0007±0.0001b | 0.0081±0.0001b | 0.0078±0.0001a | 0.0041±0.0001b |
